# The paratransgenic sand fly: A platform for control of *Leishmania *transmission

**DOI:** 10.1186/1756-3305-4-82

**Published:** 2011-05-19

**Authors:** Ivy Hurwitz, Heidi Hillesland, Annabeth Fieck, Pradeep Das, Ravi Durvasula

**Affiliations:** 1 Center for Global Health, Department of Internal Medicine, University of New Mexico, USA; 2 New Mexico VA Health Care System, Albuquerque, New Mexico, USA; 3 Rajendra Memorial Research Institute of Medical Research (RMRI), Bihar, India; 4 Department of Internal Medicine, University of Washington Medical Center, USA

## Abstract

**Background:**

*Leishmania donovani *is transmitted by the bite of the sand fly, *Phlebotomus argentipes*. This parasite is the agent of visceral leishmaniasis (VL), an endemic disease in Bihar, India, where prevention has relied mainly on DDT spraying. Pesticide resistance in sand fly populations, environmental toxicity, and limited resources confound this approach. A novel paratransgenic strategy aimed at control of vectorial transmission of *L. donovani *is presented using *Bacillus subtilis*, a commensal bacterium isolated from the sand fly gut. In this work, *B. subtilis *expressing Green Fluorescent Protein (GFP) was added to sterilized larval chow. Control pots contained larval chow spiked either with untransformed *B. subtilis *or phosphate-buffered saline. Fourth-instar *P. argentipes *larvae were transferred into the media and allowed to mature. The number of bacterial colony forming units, relative abundance and the mean microbial load were determined per developmental stage.

**Results:**

Addition of *B. subtilis *to larval chow did not affect sand fly emergence rates. *B. cere*us and *Lys fusiformis *were identified at each developmental stage, revealing transstadial passage of endogenous microbes. Larvae exposed to an exogenous bolus of *B. subtilis *harbored significantly larger numbers of bacteria. Bacterial load decreased to a range comparable to sand flies from control pots, suggesting an upper limit to the number of bacteria harbored. Emerging flies reared in larval chow containing transformed *B. subtilis *carried large numbers of these bacteria in their gut lumens. Strong GFP expression was detected in these paratransgenic flies with no spread of transformed bacteria to other compartments of the insects. This is the first demonstration of paratransgenic manipulation of *P. argentipes*.

**Conclusions:**

Paratransgenic manipulation of *P. argentipes *appears feasible. Expression of leishmanicidal molecules via commensal bacteria commonly found at breeding sites of *P. argentipes *could render adult sand flies refractory to *L. donovani *infection.

## Background

Visceral leishmaniasis (VL) in India, also known as kala azar, is caused by the parasite *Leishmania donovani *and transmitted by the sand fly *Phlebotomus argentipes*. Many rural regions in the Indian state of Bihar are endemic for VL, with conservative estimates of nearly 100,000 deaths per year attributable to this disease. For decades, control of regional epidemics of leishmaniasis has relied on spraying with DDT in areas of dense human habitation, agriculture and animal husbandry. However, rapid evolution of DDT resistance amongst target sand fly populations [1] coupled with limited resources to sustain vector eradication efforts confound this approach. Toxicity to humans, water sources, farmland and livestock renders these vector elimination strategies dangerous.

Novel approaches to control vectorial transmission of *L. donovani *are required. Our laboratory is developing paratransgenic strategies for control of *Trypanosoma cruzi *transmission by triatomine bugs as a method to reduce the human burden of Chagas disease in Latin America [2-5]. In this "Trojan Horse" approach, symbiotic gut-associated bacteria of the arthropod are transformed to express molecules with anti-parasite activity. These transformed bacteria are introduced to triatomine bugs by simulating coprophagic spread. Expression of the recombinant anti-parasite molecules within the gut of the paratransgenic vector kills *T. cruzi *and could prevent parasite transmission to humans. It is our goal to develop a paratransgenic approach to control transmission of *L. donovani *by *P. argentipes*.

Although there are no characterized symbionts within the gut of the sand fly, associations between aerobic bacteria and *P. papatasi *[6], *P argentipes and Sergentomyia *spp. [7] have been described. We recently completed a survey of aerobic bacteria of *P. argentipes *in four VL-endemic regions of Bihar, and concluded that sand fly-microbial associations reflect the environment in which the sand flies reside [8]. We identified several non-pathogenic *Bacillus *species within adult flies that are commonly deployed as soil remediation agents or cattle probiotics. Several of these species, including *Bacillus megaterium *and *Bacillus subtilis*, are generally regarded as safe, and have been utilized as microbial factories for production of recombinant proteins. We propose a paratransgenic approach to *P. argentipes *in which engineered variants of *Bacillus *spp. that express leishmanicidal molecules are delivered to sand fly larvae at natural breeding sites.

*P. argentipes *oviposits in dark corners of cattle sheds and small huts, often in association with loose, moist soil that is a mixture of humus and cow manure [9,10]. In the proposed paratransgenic approach, transformed *Bacillus *would be introduced to these breeding sites. Upon eclosure, sand fly larvae pass through four instar stages before pupation and adult emergence. Larval development occurs entirely within soil and continuous ingestion of soil material results in microbial transit within the larval gut. The mechanisms that determine whether a particular bacterial species is sequestered rather than digested during Dipteran development are not well understood. Specific, yet uncharacterized, insect-microbial interactions likely impact transstadial passage of microbes. Alternatively, it is possible that inundation of breeding soil with a large concentration of a known microbe could drive the organism through metamorphosis to the emerging adult stage. Regardless of mechanism, transstadial passage of bacteria through sand fly development is necessary for the paratransgenic strategy to succeed. If successful, the delivery of bacteria that express anti-leishmania molecules to soil-dwelling larval stages with retention and transgene expression at the adult stage would render emerging sand flies refractory to *L. donovani*, thereby disrupting the cycle of *L. donovani *transmission.

Here we demonstrate the initial proof-of-concept of the paratransgenic approach to *P. argentipes *under laboratory conditions. Green Fluorescent Protein (GFP)-expressing *B. subtilis*, when delivered to fourth instar soil larvae of *P. argentipes*, are retained in very large numbers through pupation, with constitutive gene expression in the gut lumen of emerging adult flies. This platform offers potential for field delivery of anti-leishmanial molecules to sand fly vectors of VL in regions where insecticide-based eradication efforts have failed.

## Methods

### Transformation and growth of B. subtilis

The Gram-positive-*E.coli *shuttle vector pAD43-25 was obtained from the Bacillus Genetic Stock Center. *B. subtilis *(ATCC 6051, Marburg strain) protoplasts were transformed with 1 μg of plasmid DNA using the protocol of Chang and Cohen [11]. Transformed cells were propagated on Luria Bertolini (LB) plates containing 5 μg/mL of chloramphenicol (CAM).

### Plasmid Stability in Transformed B. subtilis

One gram of sterile larval chow (a simulated sand fly breeding medium) [12] was inoculated with 6 × 10^10 ^colony forming units (CFU) of transformed *B. subtilis*, and placed at 25°C and 60-70% humidity. Over the following two weeks, a small amount of chow was removed for culture. The sample was weighed, and mixed into sterile phosphate buffered saline (PBS). Following dilution, the mixture was plated onto LB and LB+CAM plates. Plates were incubated at 37°C, and total CFU on each plate was quantified the following day. This experiment was performed in triplicate, and repeated once.

### Generation of Paratransgenic Sand Flies

In laboratory settings, sand fly larvae develop best in larval chow generated from fermented rabbit food and droppings [12]. These larvae will fail to thrive in sterile media, usually dying between the 2^nd ^and 3^rd ^instar stages. Immature larvae were therefore allowed to develop to 4^th ^instars (L4) in standard larval chow before transfer to experimental pots.

Sterile larval chow in experimental pots was spiked with untransformed and transformed *B. subtilis*. For these experiments, overnight cultures of untransformed and transformed *B. subtilis *were spun down. The resulting bacterial pellets were washed three times in PBS, and resuspended in a final volume of 6 mL in PBS. A small aliquot of each sample was removed, diluted and plated to determine cell counts. Experimental pots containing one milligram of sterile larval chow were spiked with one mL of either the untransformed (10^7 ^CFU) or transformed (10^6 ^CFU) *B. subtilis*. Twenty-five L4 larvae were added to each pot of the spiked media. As control, L4 larvae were also added to parallel pots containing sterile larval chow mixed with PBS. All experiments were performed in triplicate and repeated twice. The pots were maintained at 25°C and 60-70% humidity, and were checked daily. Sand flies were collected within 12-18 hours of emergence; larvae and pupae that failed to develop were collected on the final day (day 18) of the trial.

### Bacterial Analysis of Larvae, Pupae and E1 Flies

Larvae, pupae and emergent (E1) flies from each culture condition were individually surface sterilized for 30 sec in 70% ethanol. Each air-dried sample was homogenized in 40 μL of sterile PBS. Homogenates of each developmental stage were diluted and plated onto LB-CAM and LB media. The plates were cultured overnight at 37°C. The number of CFU for each bacterial isolate was enumerated per larva, pupa and fly. Total CFU is defined as the total number of all colonies of cultured aerobic bacteria per developmental stage. The mean bacterial load was determined by dividing the total CFU by the number of larvae, pupae or flies examined. Relative abundance of each bacterium was defined as a percentage of the total CFU of a particular bacterium divided by the total CFU.

### Identification of Bacteria from Larvae, Pupae and E1 Flies

Bacteria were initially identified using amplified ribosomal DNA restriction analysis (ARDRA) [13]. Briefly, primers B-K1/F 5'-TCACCAAGGCAACGATGCG-3' and B-K1/R1 5'-CGTATTCACCGCGGCATG-3' were used for direct colony PCR. Cells were lysed using an initial cycle step of 94°C for 2 mins. This was followed by 30 cycles of denaturation at 94°C for 20 sec, annealing at 55°C for 20 sec and extension at 72°C for 1.5 mins, with a final extension at 72°C for 2 mins. The PCR products were digested separately with Alu I (NEB) and Taq I (NEB), and were separated on 2% agarose gels. The banding pattern of each sample is compared to that of a known *B. subtilis *control. GFP expression in transformed *B. subtilis *was verified by visualization on a Zeiss AxioSkop fluorescent microscope. Amplification of GFP from cells harboring the pAD43-25 plasmid was performed using primers GFP-F 5'-TCTGTCAGTGGCGCGGGTGA-3' and GFP-R 5'-TCCATGCCATGTGTAATCCC-3'. Cells were initially lysed at 94°C for 2 mins, followed by 30 cycles of denaturation at 94°C for 20 sec, annealing at 55°C for 20 sec and extension at 72°C for 1 min. Reactions were analyzed on 1% agarose gels following a final extension at 72°C for 2 mins.

16S rDNA sequencing was subsequently used to identify the isolated bacteria. Genomic DNA was isolated using the QuickExtract bacterial DNA extraction kit (Epicentre). The 16S rDNA was amplified with the primers FD1 5'-AGAGTTTGATGGCTCAG-3' and RD1 5'-TACGGCTACCTTGTTACGACTT-3' [8]. Thermal cycling reactions consisted of an initial denaturation at 95°C for 2 mins, followed by 30 cycles of denaturation at 95°C for 30 sec, annealing at 50°C for 30 sec and extension at 72°C for 1.5 mins, followed by a single final extension at 72°C for 2 mins. Amplified products were purified on Qiagen PCR purification columns and sequenced using the PCR primers described above with the BigDye Terminator Reaction Cycle Sequencing Kit (Applied Biosystems). Bacteria were identified when their 16S rDNA sequences shared >97% homology to completed 16S rDNA sequences found in the GenBank database.

### Microscopic Analysis of E1 flies

Intact E1 sand flies were viewed at 4 × magnification using a Nikon TE2000 inverted microscope. The images were captured using a Nuance multispectral imaging system. Software associated with this system can distinguish auto-fluorescence from the desired fluorophore. Gut dissection from E1 flies was performed at Rajendra Memorial Research Institute of Medical Research, Bihar, India (RMRI) using established protocols. Gut sections were viewed under fluorescence microscopy using a Nikon Eclipse 80i microscope.

### Statistical Analysis

To detect differences in bacterial loads between each treatment group and insect developmental stage, Kruskal-Wallis one-way ANOVA tests were used. Post hoc analysis was performed using the Mann-Whitney test for Kruskal-Wallis significance. Unpaired t-tests were used to determine differences between bacterial loads from two treatment groups. A significance level of *p *= 0.05 was adopted for all analyses. GraphPad Prism 5 for Mac OS X was utilized for all biostatistical analyses.

## Results

Bacterial analysis of larvae, pupae and emergent sand flies that developed in larval chow mixed with PBS indicates that there is transstadial passage of indigenous bacterial flora (Table 1). Homogenates from every insect at the three developmental stages yielded growth on non-selection media. No growth was observed on selection media. The mean bacterial load in larvae (n = 5) was 2.8 × 10^4 ^CFU. The relative abundance of *B. cereus*, *Lys. fusiformis *and *B. subtilis *was 20%, 75% and 2.4%, respectively (Table 2). The mean bacterial load decreased slightly to 2.0 × 10^4 ^CFU in pupae (n = 9). Both *B. cereus *(relative abundance = 22%) and *Lys. fusiformis *(relative abundance = 70%) were isolated from all control pupae. *B. pumulis, B. megaterium *and *B. flexus *accounted for the remaining bacteria identified at this developmental stage. *Lys. fusiformis *(relative abundance = 67%) and *B. cereus *(relative abundance = 32%) were the predominant organisms recovered from the emergent flies (n = 4). The mean bacterial load dropped to 1.5 × 10^4 ^CFU at this stage. These numbers correlated well with previous analysis of laboratory bred sand flies (data not shown), thus no further insects were examined.

**Table 1:** Bacterial load in larvae, pupae and emergent sand flies following treatment with either 10^6 ^or 10^7 ^CFU/mg of *B.subtilis*.

Development Stage	Treatment Group	n	Mean bacterial load	+/- SD	Median bacterial load	p*
Lavae	Untreated	5	2.8 × 10^4^	2.4 × 10^4^	1.7 × 10^4^	

	10^6 ^CFU/mg	12	3.9 × 10^4^	3.3 × 10^4^	2.6 × 10^4^	

	10^7 ^CFU/mg	5	2.3 × 10^5^	1.8 × 10^5^	2.5 × 10^5^	0.055

Pupae	Untreated	9	2.0 × 10^4^	1.9 × 10^4^	1.3 × 10^4^	

	10^6 ^CFU/mg	12	6.0 × 10^4^	3.6 × 10^4^	4.6 × 10^4^	

	10^7 ^CFU/mg	12	3.3 × 10^5^	2.5 × 10^5^	2.6 × 10^5^	<0.0001

Sand fly	Untreated	4	1.5 × 10^4^	1.4 × 10^4^	1.2 × 10^4^	

	10^6 ^CFU/mg	12	2.2 × 10^4^	2.0 × 10^4^	1.7 × 10^4^	

	10^7 ^CFU/mg	12	3.9 × 10^4^	3.8 × 10^4^	2.3 × 10^4^	0.32

• Kruskal-Wallis test

The Kruskal-Wallis test indicated a slight difference in the bacterial load in larvae (*p *= 0.055) and a significant difference in pupae (*p*<0.0001). No significant difference was observed in bacterial load in emergent sand flies (*p *= 0.32). The location and identification of intergroup differences were performed using the Mann-Whitney test. In larvae, there was no difference in bacterial load between insects that developed in untreated chow and chow treated with 10^6 ^CFU/mg of *B. subtilis *(*p *= 0.60), or between insects from untreated chow and chow treated with 10^7 ^CFU/mg of *B. subtilis *(*p *= 0.055). However, statistical difference was observed in bacterial load between larvae from chow treated with 10^6 ^CFU/mg and 10^7 ^CFU/mg of *B. subtilis *(*p *= 0.035). Statistical difference were observed in pupae from untreated chow and chow treated with 10^6 ^CFU/mg of *B. subtilis *(*p *= 0.0062), pupae from untreated chow and chow treated with 10^7 ^CFU/mg of *B. subtilis *(*p *< 0.0001) and pupae from chow treated with 10^6 ^and 10^7 ^CFU/mg of *B. subtilis *(p < 0.0001).

**Table 2:** Relative abundance of *B.subtilis, B. cereus and Lys. fusiformis *in larvae, pupae and sand flies reared in PBS-treated chow (control) or chow spiked with *B. subtilis*

	PBS-treated chow			***B***. ***subtilis *spiked chow**		
	***B***. ***subtilis***	***B***. ***cereus***	***Lys fusiformis***	***B***. ***subtilis***	***B***. ***cereus***	***Lys fusiformis***

**Larvae**	2.4%	20%	75%	99%	0.9%	

**Pupae**		22%	70%	94%	2.1%	

**Sand flies**	0.6%	32%	67%	74%	25%	1%

The relative abundance of each bacterium was defined as the percentage of the total CFU of each bacterium divided by the total CFU per developmental stage. *B. cereus *and *Lys fusiformis *persisted through pupation with little to no loss in relative abundance in insects that were allowed to develop in PBS-treated chow. Although both organisms are displaced by the exogenous *B. subtilis*, they were still present in the emergent sand flies. Exogenous *B. subtilis *was able to displace most endogenous bacteria in the larval and pupal stages of growth.

When insects were allowed to develop in chow inoculated with 10^7 ^CFU of untransformed *B. subtilis*, the bacterium was recovered from all larvae (n = 5), pupae (n = 12) and flies (n = 12) on non-selection media (Table 2). No growth was observed on selection media from any of these specimens. The relative abundance of *B. subtilis *was 99% and 94% in larvae and pupae, respectively, but dropped to 74% in the emergent flies. The remaining microbes in the emergent flies were identified to be *B. cereus *(25%) and *Lys. fusiformis *(1%). The mean microbial load in larvae was 2.3 × 10^5 ^CFU, increased to 3.3 × 10^5 ^CFU in pupae, and dropped to 3.9 × 10^4 ^CFU in emergent flies. We were only able to recover 5 larvae at the conclusion of this treatment.

**Figure 1:**
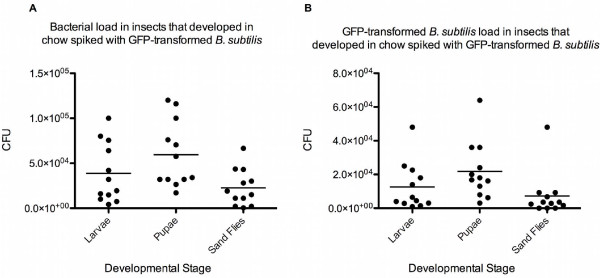
**Bacterial load in larvae, pupae and sand flies from chow inoculated with GFP-transformed *B. subtilis***. There is an average increase of 34% in CFU count when homogenates from each developmental stage were plated onto non-selection (A) versus selection (B) media. The increase in CFU count on non-selection media appears to correlate well with the calculated rate of plasmid loss for *B. subtilis *for this system.

When larvae were allowed to develop in sterile chow that was inoculated with 10^6 ^CFU of GPF-expressing *B. subtilis*, the transformant was the only bacterium recovered from homogenates of all larvae (n = 12), pupae (n = 12) and 75% of flies (n = 12) plated on selection medium. The remaining E1 flies did not harbor any aerobic microbes. The mean number of transformed *B. subtilis *isolated from larvae was 1.3 × 10^4 ^CFU. This increased slightly to 2.2 × 10^4 ^CFU during pupation, and dropped to 7.2 × 10^3 ^CFU in the emergent fly. (Figure 1B). There was no horizontal transfer of the pAD43-25 plasmid to any other bacteria as all cells growing on selection media were identified to be *B. subtilis*.

There is approximately a 34% increase in CFU count when the homogenates from the above experiment were plated onto non-selection medium (Figure 1A). The mean CFU count was determined to be 3.9 × 10^4^, 6.0 × 10^4 ^and 2.2 × 10^4 ^from larvae, pupae and emergent flies, respectively. As expected, *B. subtilis *was identified to be the predominant microbe in the larval and pupal homogenates. However, the relative abundance of *B. subtilis *dropped to 26% in the emergent sand fly. The remaining bacteria in the sand flies were identified to be *B. cereus*, *Ly. fusiformis *a number of other *Bacillus *spp, *Pseudomonas *spp, and *Staphylococcus *spp. Fluorescent microscopy and GFP-specific PCR analysis suggest that there was no horizontal transfer of the pAD43-25 plasmid to any of these other bacteria (data not shown). However, a portion of the *B. subtilis *colonies examined did not fluoresce, and were PCR negative for the GFP gene. Since the relative abundance of endogenous *B. subtilis *in emergent flies was only 0.6%, these results would suggest plasmid loss.

**Figure 2:**
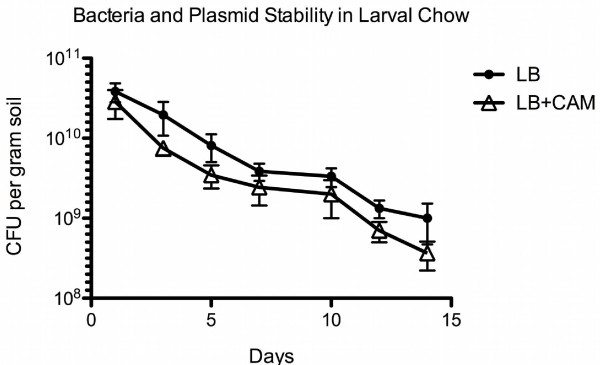
**Plasmid stability of transformed *B. subtilis *in larval chow**. The viability of the transformed cells shows a steady decline in larval chow. At the end of the two-week experimental period, close to 40% of the remaining viable *B. subtilis *no longer confer antibiotic resistance, suggesting plasmid loss.

To determine plasmid stability in larval chow the *B. subtilis *transformant was inoculated into larval chow in the absence of antibiotic selection. As shown in Figure 2, there was a 97.5% decrease in *B. subtilis *CFU's over the two-week period. At the end of the two weeks, the number of cells recovered on non-selection medium was 2.5% (1 × 10^9 ^cells/gram) of the original inoculum, while the number of CFU's recovered on selection plates was 1% (3 × 10^8 ^cells/gram). The rate of plasmid loss was calculated to be approximately 40% over a two-week period. This would support the 34% increase in *B. subtilis *CFU's from larvae and pupae growing on non-selection medium.

The bacterial load at each developmental stage was compared across all treatment groups (Table 1). Larvae and pupae isolated from chow spiked with 10^7 ^CFU of bacteria/mg appear to harbor significantly more bacteria than stages that developed in control chow and in chow spiked with 10^6 ^CFU/mg of *B. subtilis*. However, there is no significant difference in bacterial load in emergent flies, suggesting that there may be an upper limit to bacterial carriage at this stage.

**Figure 3:**
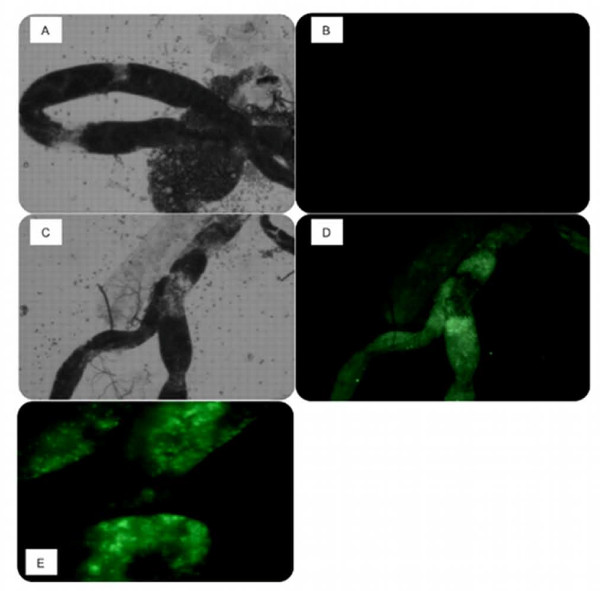
**Bright field and fluorescent micrograph of *P. argentipes *midgut**. Midgut of emergent *P. argentipes *exposed to wild-type *B. subtilis *as 4th instar larvae at 10×, A: bright field, B: fluorescence. Midgut of E1 *P. argentipes *exposed to pAD43-25-transformed *B. subtilis *as 4th instar larvae at 10×, C: bright field, D: fluorescence, E: fluorescence at 40×.

**Figure 4:**
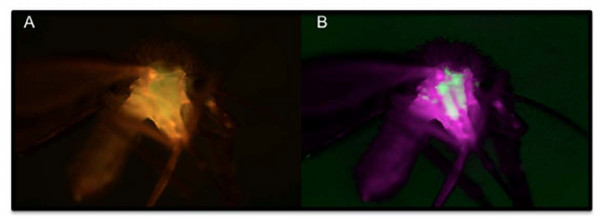
**Whole mount of paratransgenic sand fly micrograph**. A: shows the auto-fluorescence associated with the outer carapace and specific GFP fluorescence within the sand fly. B: shows GFP-specific fluorescence signal uncoupled from the background. These 4× -images were captured using a Nuance multispectral imaging system. GFP-specific fluorescence is contained to the midgut chamber of the adult sand fly with no evidence of transfer to other regions of the insect.

The midguts of emergent paratransgenic sand flies were dissected and examined with fluorescence microscopy. Strong GFP signals were visualized throughout the midgut of all emergent flies (Figure 3C and 3D), suggesting the expression of a functional protein. No fluorescent signals were detected from sand flies that developed in PBS-treated chow (Figures 3A and 3B). Whole mounts of paratransgenic sand flies showed strong green fluorescence in the thorax of the insect (Figure 4) where the midgut is located. No fluorescence was detected outside of the insect midgut, suggesting sequestration of transformed bacterial in this compartment only.

**Figure 5:**
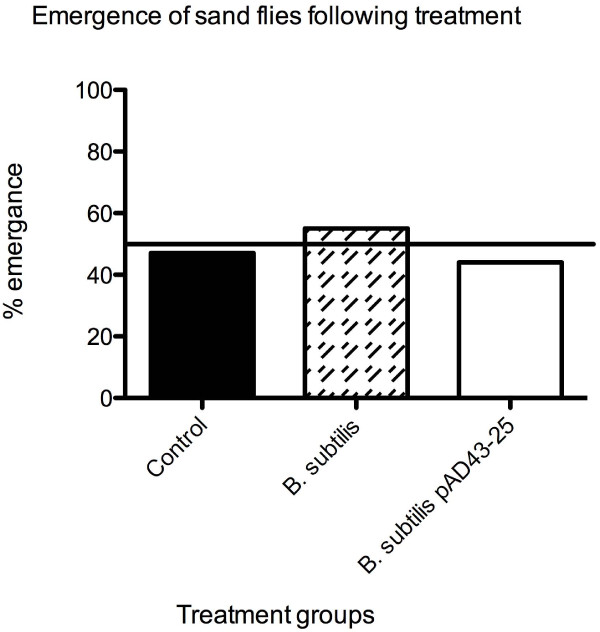
**Addition of *B. subtilis *to the larval chow had no effect on maturation**. Under optimal insectary conditions, only 50% of larvae undergo full metamorphosis (K. Ghosh, personal communication). This percentage is reflected in our study.

For this approach to be successful, the uptake of large amounts of *B. subtilis *by sand fly larvae should not significantly reduce rates of maturation. We monitored sand fly emergence over a period of 18 days, and determined that complete metamorphosis in *B. subtilis*-treated larvae is similar to that of control larvae (Figure 5). This development rate of approximately 50% is comparable to that observed by other investigators in insectary settings (K. Ghosh, personal communication).

## Discussions

Here, we demonstrate, under laboratory conditions, paratransgenic manipulation of *P. argentipes*, the sand fly vector of *L. donovani*. As a platform for expression of foreign genes in disease-transmitting adult flies, we first demonstrated the transstadial passage of indigenous flora from fourth instar sand fly larvae to the emergent adult sand fly. We further showed that indigenous flora could be displaced with a complementary microbe, such as GFP-expressing *B. subtilis*, to generate paratransgenic sand flies. *B. subtilis *was identified in our previous study of field caught sand flies from endemic VL regions in Bihar [8]. This organism is generally regarded as safe [14], has probiotic activities [15], is utilized in soil remediation [16] and is highly amendable to genetic manipulation [17]. *B. subtilis *was present at very low background levels in the control insects examined in this study. The addition of large numbers of *B. subtilis*, up to 10^7 ^CFU/mL, to larval chow did not affect sand fly eclosure. Although emergent paratransgenic sand flies appeared phenotypically normal, they harbored a large number of bacteria, up to 10^4 ^CFU, on the day of emergence. In the current study, all sand flies were examined within one day of eclosure. At this time point, the bacterial load in sand flies that emerged from PBS-spiked or *B. subtilis *spiked chow were not significantly different (Figure 1), suggesting an upper limit to colonization. Field collections of *P. argentipes *in Bihar revealed low levels of *B. subtilis *(10^1 ^- 10^2 ^bacteria/fly) [8]. The ages of the field-caught sand flies were not determined in our previous work. It is possible that the number of bacteria decreases as the sand flies mature. Studies are currently underway to examine this hypothesis. Deleterious phenotypes with reduced fitness due to paratransgenic interventions would likely result in rapid selection against paratransgenic insect populations. In our studies with paratransgenic triatomine bugs, we have verified that engineered symbiotic bacteria exert no negative fitness effects [3]. Experiments that explore the potential impact of transformed *B. subtilis *on the survival and fecundity of *P. argentipes *experiments are currently in progress.

The average life span of female *P. argentipes *is about 12 days. During this time, the insect takes, on average, two blood meals. Amastigote-infected macrophages are acquired during the first blood meal at about day 2-3 post-emergence [18]. When released into the mid-gut of the sand fly, the amastigotes differentiate into promastigotes. These infective forms of the parasite are subsequently transmitted at the second blood meal, at about day 7-8, after oviposition. For the paratransgenic strategy to be successful in this system, the engineered *B. subtilis *should persist within the sand fly gut until the first blood meal. However, an anti-leishmania recombinant molecule may remain active in the sand fly gut in the absence of recombinant carrier. In our experience with paratransgenic triatomine bugs, recombinant cecropin A persisted for over 6 months in the gut lumen with biological activity [3]. Such an outcome would be viewed as highly desirable since the intended biological effect would occur with reduced risk of unwanted spread of transgenic bacteria via activities of the adult flies. Studies are currently underway to determine the persistence of transformed *B. subtilis *in the adult sand fly, whether carriage of these bacteria would have a detrimental effect on longevity and fecundity of the paratransgenic sand flies and if there is maternal transmission of these genetically modified bacteria to the following generation.

Large populations of *P. argentipes *are intimately associated with human residence in many VL-endemic regions of Bihar, India. For decades, the control of regional epidemics of leishmaniasis had relied on spraying with DDT in areas of dense human habitation, agriculture and animal husbandry. Rapid evolution of DDT resistance amongst target sand fly populations [1] coupled with limited resources to sustain vector eradication efforts has confounded this approach. Toxicity to humans, water sources, farmland and livestock further detract from vector elimination strategies. Nevertheless, in February of 2009, another major campaign was launched to blanket 21 districts of Bihar State with DDT. Surveys of *P. argentipes *in highly endemic regions of the state reveal DDT resistance rates that approach 50-65 percent (V. Kumar, personal communication). Continued efforts to eradicate sand flies that are inextricably linked to human activities in the face of an escalating epidemic of drug-resistant *L. donovani *appear increasingly futile [19].

The breeding sites of *P. argentipes *are fairly well defined in several VL-endemic regions. Sampling of *P. argentipes *larvae in the Indian states of West Bengal and Bihar revealed abundance of immature stages in dark corners of cattle sheds and small huts, often in association with loose, moist soil that is a mixture of humus and cow manure [9,10]. The engineered *B. subtilis *could be delivered to known soil breeding sites of *P. argentipes *throughout Bihar State. By utilizing an environmental bacterium and understanding the developmental life cycle of *P. argentipes*, we have developed a potentially powerful, safe and inexpensive methodology for the control of parasite transmission.

Bacterial populations acquired by Dipteran larvae decrease in numbers and are often lost during metamorphosis. However, transstadial passage of microbes in true flies (Diptera) has been described. Strains of *E. coli *and other microbes transit from larval to adult stages of the house fly (Diptera: Muscidae) [20-22]. Transformed *Pantoea stewartii *fed to mosquito larvae (Diptera; Culicidae) survived to pupation, but not beyond [23]. Though transstadial passage of bacteria from larvae to adult sand flies has not previously been demonstrated, Gram-negative bacteria have been isolated from the gut of fourth-instar larvae, pupae and newly emerging females of the fly, *P. duboseqi *(Diptera: Psychodidae) [24].

Sand fly larvae were transferred to an aseptic environment at the L4 stage. These larvae were, therefore, populated with environmental bacteria before transfer to the experimental chambers. Two prominent isolates from control insects were *B. cereus *and *Lys fusiformis*. Although the majority of these two bacteria were displaced by large inocula of exogenous *B. subtilis *in larval and pupal stages, the remaining population persisted through pupation. In the control group of *P. argentipes*, the relative abundance of *B. cereus *remained at approximately 20% through all developmental stages. *B. cereus *is a common soil-dwelling microbe. While several strains have probiotic activities, others are associated with food-borne illnesses, rendering them unfit for paratransgenic applications. *Lys. fusiformis *is a spore-forming, Gram-positive rod-shaped bacterium isolated from soil [25]. This bacterium is related to *Lys. sphaericus*, a microbe that is known to kill mosquitoes [26], but as demonstrated in this study, is not pathogenic to insects. Furthermore, *Lys fusiformis *is not a known human pathogen, suggesting its suitability for paratransgenic applications. Avenues to transform this organism are currently being explored.

## Conclusions

This study demonstrates paratransgenic manipulation of the sand fly vector of kala azar, *P. argentipes*, under laboratory conditions. The use of an environmental commensal bacterium for delivery of foreign genes to developmental stages of the sand fly serves as a platform to consider paratransgenic approaches in field conditions as a tool to control vectorial transmission of *L. donovani*. In other studies, we have shown that the anti-microbial peptide, mellitin, exerts potent activity against promastigote forms of *L. donovani *at micro-molar concentrations (H. Hillesland et al, unpublished data). It is interesting to note that *L. donovani *undergo transformation from amastigotes to dividing and infectious promastigotes at the sand fly midgut. The targeted delivery of leishmaniacidal molecules by a commensal bacterium within this region of the adult sand fly would disrupt this developmental transition, thereby generating paratransgenic sand flies that are refractory to *L. donovani *infection. This approach could be highly advantageous in the battle to decrease the burden of visceral leishmaniasis in India.

## Abbreviations

ARDRA: amplified ribosomal DNA restriction analysis; CAR: carbenicillin; CFU: colony forming units; GFP: green fluorescent protein; LB: Luria Bertolini; PBS: phosphate buffered saline; VL: visceral leishmaniasis

## Competing interests

The authors declare that they have no competing interests.

## Authors' contributions

IH, HH and AF designed the experiments in collaboration with the other authors. HH collected and initiated the processing of the insects, as well as drafted the first version of this manuscript. IH completed the processing of the insects, analyzed and interpreted the results, and revised and finalized the manuscript in consultation with the other authors. PD provided the insects for this study. RD supervised the design and implementation of this study. All authors revised and approved the final version of this manuscript.
